# Faster turnover of taxonomic over functional bacterial composition during vermicomposting indicates increasing functional redundancy

**DOI:** 10.1371/journal.pone.0354276

**Published:** 2026-07-22

**Authors:** Manuel Aira, Marcos Pérez-Losada, Keith A. Crandall, Jorge Domínguez

**Affiliations:** 1 Departamento de Ecoloxía e Bioloxía Animal, Grupo de Ecoloxía Animal (GEA), Universidade de Vigo, Vigo, España; 2 Department of Biostatistics and Bioinformatics, Computational Biology Institute, Milken Institute School of Public Health, George Washington University, Washington, District of Columbia, United States of America; Duy Tan University: Dai Hoc Duy Tan, VIET NAM

## Abstract

Microbial taxonomy underpins microbial functionality; consequently, changes in taxonomic composition are expected to influence community functional profiles. However, functional redundancy—the ability of different microorganisms to perform similar functions—can sometimes decouple taxonomic and functional variation in microbial communities. In this study, we estimated the turnover rates of bacterial taxonomic and functional composition, as well as functional redundancy, across different substrates during vermicomposting. Our results showed that, during the early stages of vermicomposting, bacterial functional composition largely mirrored taxonomic composition across substrates. However, by the final stage, samples converged toward similar functional profiles despite remaining taxonomically distinct, indicating an increase in functional redundancy over time. Consistent with this pattern, the taxonomic turnover rate was approximately twelve times higher than the functional turnover rate. Functional redundancy increased throughout the process, with values ranging from 0.27 to 0.58 (on a scale from 0 to 1). The high turnover rates observed demonstrate that vermicomposting is an accelerated decomposition system with respect to both bacterial taxonomic and functional dynamics, exhibiting turnover rates more than 200 times higher than those reported for soil ecosystems. However, because this study did not include a non-earthworm control treatment, the accelerated turnover rates cannot be attributed exclusively to earthworm activity and should instead be interpreted as the result of the combined effects of earthworms and microorganisms. Future studies should incorporate non-worm controls to disentangle the specific contribution of earthworms and to evaluate how varying earthworm densities influence microbial turnover dynamics.

## Introduction

Microbial communities exhibit a vast taxonomic diversity, which is crucial to the fundamental functions they perform in supporting biogeochemical cycles [1]. Further, microbial functionality depends on taxonomy, meaning that certain functions can only be performed by specific microbial taxa, whereas others are shared by many microorganisms [2]. Functional redundancy is a characteristic of microbial communities and implies that different microorganisms share the same genes of a specific metabolic pathway allowing different species to perform similar functions [2].

Advancements in next-generation sequencing technologies and time-series analysis have enabled the study of the temporal dynamics of microbial communities revealing complex patterns during succession in the sea, soil, and decomposition processes [3–6]. However, research studying community turnover rates (i.e., the amount of change over time in both taxonomic and functional microbial composition) is still lacking. One method of calculating turnover rates involves measuring the dissimilarity in taxonomic and functional composition of the bacterial communities and modeling it over time [7]. During microbial succession, functional redundancy may decouple taxonomic and functional turnover rates, with high redundancy leading to relatively low functional turnover despite high taxonomic turnover [2].

During vermicomposting, earthworms and microorganisms work together to convert organic waste into vermicompost through two steps [8]. First, earthworms digest microbial communities of organic waste egesting them as casts as a consequence of the gut associated processes (GAPs). After being released, casts undergo cast-associated processes (CAPs) [8], which include aging processes and interactions with non-processed material, ultimately resulting in vermicompost. As result of GAPs and CAPs, microbial communities of cast and vermicompost differ widely from those of unprocessed material [3,9–14].

Recent studies indicate that the majority of bacteria found in earthworm casts are of gut origin rather than of ingested-substrate origin [9,15]. This suggests that the bacterial composition of vermicompost is mainly determined by the earthworm digestion of raw substrates. This is because earthworms are expelling their gut microbiomes. After defecation, the bacterial succession on egested casts is largely determined by the chemical composition of the substrates [8]. Recent studies indicate that vermicompost made from different materials contain a diversity of bacterial taxa. This diversity is influenced by the composition of the raw substrates, i.e., more similar substrates resulted in more similar bacterial communities [3,9–14]. Given that microbial function is linked to microbial taxonomy, this similarity in taxonomy should be reflected in functional composition. This, in turn, affects the biological properties of vermicompost, i.e., the production of antibiotics, plant hormones and nutrient mineralization, as recent studies have shown [3,9–14]. However, we still do not know the rates of taxonomic and functional turnover, and whether they are substrate-dependent. Additionally, it is also unclear whether taxonomic and functional turnover occur at similar or different rates, which may depend on the degree of functional redundancy in bacterial communities.

In this study, we describe how vermicomposting affects the taxonomic and functional composition of bacterial communities grown in different substrates using new and available next-generation sequence data. While previous research on the microbial dynamics of vermicomposting has largely been descriptive, documenting structural shifts in microbial succession during vermicomposting, our study shifts the focus toward understanding the underlying ecological dynamics. To bridge this knowledge gap, this study quantifies the explicit taxonomic and functional turnover rates of bacterial communities over time. Additionally, we explore the role of functional redundancy, a critical yet understudied factor, to establish how this metabolic buffering influences community stability and relationships during organic waste stabilization. We hypothesize that if functional redundancy is absent, taxonomic and functional turnover rates should be similar, whereas if functional redundancy is present, these turnover rates should differ, with taxonomy showing higher turnover rates compared to functional composition.

## Materials and methods

### Vermicomposting sampling design

We combined vermicomposting data from vermireactors fed with grape marc from white [12] (Albariño variety), red [10] (Mencía variety) and distilled white grapes [11], silver wattle [13] (*Acacia dealbata*), scotch broom [3] (*Cytisus scoparius*), seaweeds [14] (desalinized mix of *Ulva lactuca*, *Ascophyllum nodosum* and *Fucus spiralis* in equal proportions) and coffee grounds. In all vermireactors, we established vermicomposting systems using the earthworm species *Eisenia andrei*, as described previously [3], and measured the earthworm population density at regular intervals in each vermicomposting trial. Vermicomposting was performed in a rectangular metal pilot-scale vermireactor (4 m long × 1.5 m wide × 1 m high). The vermireactor was housed in a greenhouse with no temperature control. In each sampling time we collected five samples from the substrate layer in the vermireactor using a core sampler. To do this we divided the substrate layer into 5 equal sections, and randomly took five samples from each section. The reactor that was fed with coffee grounds had the highest density of earthworms overall (10,527 ± 549 earthworms m^-2^), while the reactors that were fed with distilled grape marc had the second highest density (1,669 ± 136 earthworms m^-2^), followed by the reactors fed with seaweed (851 ± 33 earthworms m^-2^), scotch broom (834 ± 77 earthworms m^-2^), silver wattle (642 ± 46 earthworms m^-2^), Albariño grape marc (451 ± 33 earthworms m^-2^) and Mencía grape marc (270 ± 11 earthworms m^-2^). Vermireactors also showed variation in processing times. Vermireactors fed with scotch broom along with Albariño and Mencía grape marc took 91 days to process, while vermireactors fed with silver wattle and coffee grounds took 56 days to process. Lastly, vermireactors fed with distilled grape marc and seaweed took 42 and 28 days, respectively, to complete the process.

We did not include controls (i.e., reactors without earthworms) because our goal was to compare how the different substrates may affect the rate of temporal taxonomic and functional turnover as well as functional redundancy.

### High-throughput sequencing and analysis of 16S rRNA amplicons

DNA was extracted using the MO-BIO PowerSoil® kit following the manufacturer’s protocol. All laboratory procedures were performed under a laminar flow hood to prevent contamination of the samples with microorganisms from the surrounding environment. We amplified and sequenced a fragment of the 16S rRNA gene covering the V4 region with a dual-index sequencing strategy using an Illumina MiSeq [16]. To process raw sequences, we used usearch v11 [17] and vsearch v2.17.1 [18]. First, forward and reverse reads were merged and filtered with usearch fastq_mergepairs (-relabel @ -fastq_maxdiffs 10 -fastq_pctid 10 -fastq_minmergelen 250 -fastq_maxmergelen 256) and vsearch fastq_filter (fastq_maxee 1.0) commands, respectively. Sequences were then dereplicated with vsearch derep_fulllength command (default settings). Finally, we used usearch unoise3 command (default settings) to infer the zero radius OTUs (zOTUs, analogues of DADA2 ASVs) and remove chimeras [19]. We estimated a zOTU abundance table using vsearch usearch_global command using the reads before quality filtering with a minimum fractional identity of 0.97 to improve sensitivity and to correct sequencing and PCR errors [20]. Representative sequences for each zOTU were classified with mothur [21] against the SILVA v138.1 database. We discarded any sequences classified as Chloroplast, Mitochondria, Archaea and Eukaryota, and remove any bacterial sequences not classified to the Phylum level (0.36% of sequences). Our data cleaning efforts resulted in 7,993,718 sequences distributed among 11,282 zOTUs and 188 samples. Sequences were upload to GenBank SRA database under accessions PRJNA772065 (silver wattle), SRP171648 (Albariño grape marc), PRJNA555188 (Mencía grape marc), SRP120990 (scotch broom), PRJNA602410 (distilled grape marc), PRJNA1069356 (seaweeds) and PRJNA1069362 (coffee grounds).

### Statistical analysis

We further processed the zOTU table with the LULU algorithm with default settings to reduce taxonomic redundancy [22]. LULU curated the zOTU table based on sequence identity and co-occurrence patterns and kept 6,812 zOTUs from the initial 11,282 zOTUs. Since raw substrates cannot be considered earthworm worked substrates, we removed them from all analyses retaining 154 samples and 6,676 zOTUs. After that we did a prevalence filtering on our data keeping only zOTUs present in at least 10% of the samples; this removed 37% of the zOTUs, but only 2% of the sequences, retaining 4,145 zOTUs and 6,505,589 sequences (42,244 ± 23,920 mean ± standard deviation of sequences per sample). We rarefied the data to a minimum of 12,562 sequences/sample resulting in 4,137 zOTUs.

The functional composition of the metagenomes was inferred using the Phylogenetic Investigation of Communities by Reconstruction of Unobserved States software package (PICRUSt2) [23]. We followed the procedure described at https://github.com/picrust/picrust2/wiki/Full-pipeline-script using the LULU zOTUs filtered dataset. The weighted nearest sequenced taxon index (NSTI) for our samples was 0.124 ± 0.005 (mean ± s.e.), which is considered acceptable for samples from relatively underrepresented or poorly characterized environments [23]. We also calculated the metagenome contributions, i.e., which functions coded each zOTU, passing the –stratified option to the PICRUSt2 run.

We used the LULU zOTUs (filtered and rarefied) and the KEGG Orthology (KO) datasets for taxonomic and functional analysis, respectively. Prior to analysis, we transformed taxonomic and functional matrices from abundance to presence-absence; this allowed for taxonomic and functional community turnover to be interpreted as species replacement over time [7]. To estimate the functional redundancy of bacterial communities, we used the metagenome-contributions table from PICRUSt2 and for each zOTU. We determined the total number of KOs and calculated the functional redundancy over that variable using the method of Royalty and Steen [24]. This method measures how evenly different species contribute to a community-aggregated trait—a trait assessed at the community level and divided among species. The resulting functional redundancy index varies from 0 (where only one species contributes to the trait; no redundancy) to 1 (where all species contribute equally; maximum redundancy).

Following the procedure outlined in Martinović et al. [7], we looked for directional, non-directional, and stochastic temporal variation over time. To differentiate among these trends, we performed PERMANOVA analyses with time consider as a continuous (linear effect) or categorical (non-linear effect) variable. Lack of time effect would indicate stochastic temporal variation, whereas a significant linear effect would reveal a directional time trend. Further, since a categorical effect includes the possibility of a linear effect, categorical year would yield similar explanatory power (measured as R^2^) in presence of a directional effect [7]. Higher R^2^ in categorical analysis would indicate non-linear temporal trends. We carried out PERMANOVA analyses for each vermicomposting substrate using the adonis2 function from the vegan library [25] with 999 permutations and controlling for each sampling replicate separately. Moreover, to ease the visualization of taxonomic and functional composition during vermicomposting, we applied principal coordinate analysis using the Sorensen dissimilarity index.

We quantified the rate of community turnover over time using the Sorensen dissimilarity between the samples of each reactor during vermicomposting. Sorensen values range between 0 and 1, so can be read as percent differences in community dissimilarity. Then, for each vermicomposting reactor, we fitted linear mixed effect models as implemented in the nlme package [26], using the Sorensen taxonomic and functional dissimilarities as response variables and temporal distance between samples as predictor variables. The effect of time nested in each sample was considered as a random factor to account for non-independence of samples due to repeated measures (random = ~1|time_cont/site). In this model, the slope indicates the rate of overall temporal turnover measured in Sorensen units per day [7]. Using this procedure, time is measured as a distance in days between sampling times. For example, for a vermireactor with sampling times of 7, 14, 28, 42 and 91 days, we have distances of 7, 14 (two measures), 21, 28, 35, 49, 63, 77 and 84 days. We used the same model to analyse the effect of time on functional redundancy, although in this case, time was measured as days and not distance in time. We then computed bootstrap confidence intervals (1,000 replicates) for the two regression parameters (intercept and slope) using the bootstrap function from the lmeresampler library [27]. Thus, the null hypothesis of no temporal turnover for each variable (taxonomic and functional composition and functional redundancy) across the seven vermireactors was rejected when the 95% confidence interval of the slope did not overlap zero. This procedure was also done with a model in which we included all substrates in the analysis of temporal taxonomic and functional turnover rate and functional redundancy. We could not include earthworm density as a covariate in any analysis (by substrate or joining all substrates) because it was estimated at different times than those used to get samples for metagenomics analysis (i.e., metagenomics and earthworm density sample times did not match).

We analysed and plotted all the data using the phyloseq [28], ggplot2 [29], ggeffects [30] and patchwork packages [31] (Pedersen, 2020) using R version 4.0.3 [32].

## Results

**Fig 1:**
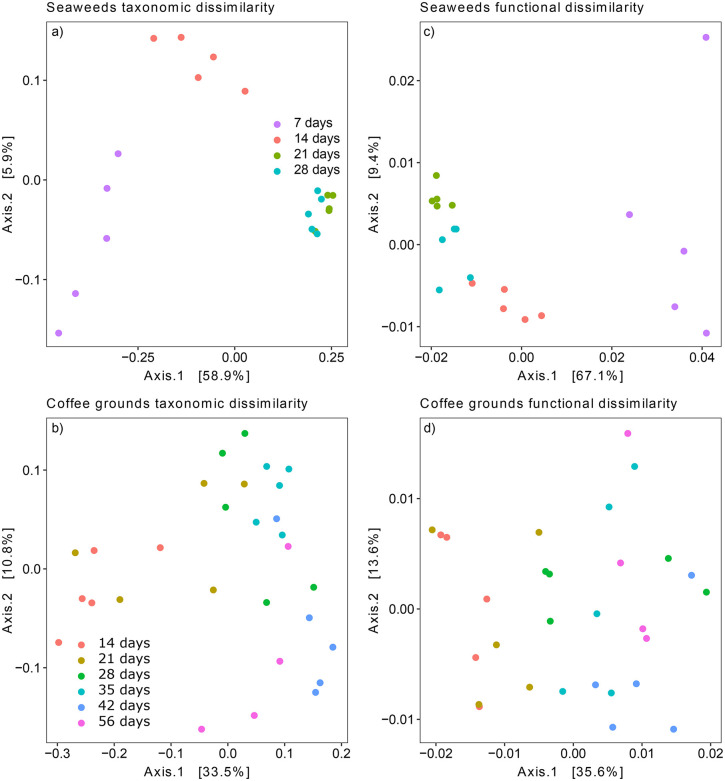
PCoA ordination plots showing the changing patterns in taxonomic (a,b) and functional (c,d) composition of bacterial communities during vermicomposting of seaweed and spent coffee grounds.

Vermicomposting significantly modified the taxonomic and functional composition of substrates over time, as shown by PERMANOVA analyses (Table 1). When time was considered as a categorical factor its effect on both taxonomic and functional community turnover was consistently larger than when considered as a continuous factor, even when analysing all substrates together (Table 1). This suggests that non-linear time effects were more prevalent than linear effects during vermicomposting. On average, there was a 72% and 80% increase in both taxonomic and functional turnover, respectively; these values were over 100% when considering all substrates together (Table 1). Non-linear temporal trends were evident for both taxonomic and functional community turnovers in the PCoA plots (Fig 1, S1-S2 Figs). When considering taxonomic and functional community turnover, samples from earlier time points in vermireactors always clustered separately from those taken at later stages. However, unsegragated clustering patterns were observed in vermireactors that were fed with coffee grounds and distilled grape marc in both taxonomic and functional composition across most samples (Fig 1, S1-S2 Figs).

**Table 1:** The effect of time (as continuous or categorical variable) on taxonomic (bacterial zOTUs) and functional (KOs) composition during vermicomposting of different substrates analysed by PERMANOVA with 999 permutations (df, degrees of freedom).

Substrate	Measure	Time (days)	df	R^2^	*P*
All substrates	taxonomic	continuous	1, 153	0.06	0.001
		categorical	8, 153	0.14	0.001
	functional	continuous	1, 153	0.09	0.001
		categorical	8, 153	0.18	0.001
Albariño grape marc	taxonomic	continuous	1,24	0.46	0.001
		categorical	4,24	0.81	0.001
	functional	continuous	1,24	0.52	0.002
		categorical	4,24	0.80	0.001
Mencía grape marc	taxonomic	continuous	1,19	0.40	0.001
		categorical	3,19	0.67	0.001
	functional	continuous	1,19	0.37	0.001
		categorical	3,19	0.63	0.001
Distilled grape marc	taxonomic	continuous	1,24	0.12	0.001
		categorical	4,24	0.30	0.001
	functional	continuous	1,24	0.10	0.008
		categorical	4,24	0.32	0.001
Seaweed	taxonomic	continuous	1,19	0.47	0.001
		categorical	3,19	0.62	0.001
	functional	continuous	1,19	0.48	0.001
		categorical	3,19	0.62	0.001
Coffee grounds	taxonomic	continuous	1,28	0.21	0.001
		categorical	5,28	0.44	0.001
	functional	continuous	1,28	0.24	0.001
		categorical	5,28	0.45	0.001
Scotch broom	taxonomic	continuous	1,14	0.54	0.001
		categorical	2,14	0.72	0.003
	functional	continuous	1,14	0.57	0.001
		categorical	2,14	0.72	0.002
Silver wattle	taxonomic	continuous	1,19	0.49	0.001
		categorical	3,19	0.68	0.001
	functional	continuous	1,19	0.33	0.001
		categorical	3,19	0.56	0.001

**Fig 2:**
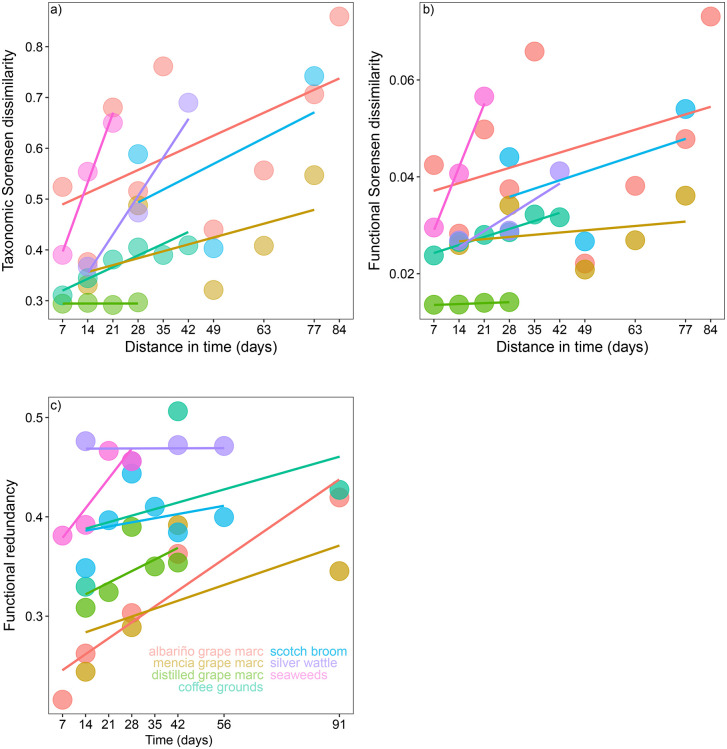
Patterns of temporal changes in the dissimilarity of taxonomic (a) and functional composition (b), and functional redundancy (c) during vermicomposting of white (Albariño), red (Mencía) and distilled grape marc, spent coffee grounds, scotch broom, silver wattle and seaweed. Data shown represent mean estimates per substrate and time (see S3, S4 and S5 Figs for plots with all sampling points). Lines show the mixed-model fit. Note that each vermicomposting reactor had different measures of time. The intercepts represent the baseline community dissimilarity or functional redundancy when temporal distance equals zero, and the slopes represent the overall rates of taxonomic and functional turnover or functional redundancy.

Vermicomposting led to an increase in taxonomic dissimilarity over time, with turnover rates that were characteristic of each substrate (Fig 2a, S3 Fig, Table 2, S1 Table). Reactors fed with silver wattle and seaweed exhibited steeper slopes than those fed with other substrates, indicating a 3.1-fold (silver wattle) and 6.1-fold (seaweed) increase in taxonomic turnover rates (Fig 2a, Table 2, S1 Table). The baseline dissimilarity between vermicomposting processes, which measures how different the samples were when earthworms started to process the substrates, varied depending on the substrate. Thus, the vermireactors fed with Albariño grape marc and scotch broom had intercepts 1.4 times higher than those fed with other substrates (Fig 2a, Table 2, S1 Table). All slopes and intercepts, except for the vermireactor fed with distilled grape marc, were statistically significant, which indicates, in the case of slopes, temporal turnover for taxonomic composition (Table 2, S1 Table). When analysing all substrates together the overall temporal turnover rate decreased to values similar to those of vermireactors fed with Albariño grape marc and coffee grounds (time estimate = 0.0035, 95% CI = 0.003, 0.004), with a baseline dissimilarity value of 0.481 (95% CI = 0.296, 0.663).

**Table 2:** Estimates of the time and intercept of linear mixed-effect models for vermicomposting of each substrate, 95% confidence interval after 1,000 bootstrap replicates, relating community dissimilarity and distance in time of taxonomic and functional composition, and functional redundancy. The times and intercepts represent the overall rates of turnover and the reference point community dissimilarity and functional redundancy when distance in time equals zero. Please note that functional redundancy was calculated per sampling time (day) and not distance in days. The null hypothesis of no temporal turnover for each parameter (intercept and time) and type of measurement (taxonomic and functional composition and functional redundancy) across the seven substrates was rejected when the 95% confidence interval of the time did not overlap zero.

		Taxonomic composition		Functional composition		Functional redundancy	
Substrate	Term	Estimate	95% CI	Estimate	95% CI	Estimate	95% CI
Albariño grape marc	Intercept	0.4797	0.4347, 0.5255	0.0398	0.0314, 0.0481	0.3009	0.282, 0.3189
	time	0.0035	0.0026, 0.0044	0.0003	0.0001, 0.0005	0.0024	0.0019, 0.0028
Mencía grape marc	Intercept	0.4051	0.3395, 0.4717	0.0311	0.0258, 0.0364	0.3541	0.3153, 0.3919
	time	0.0018	0.0005, 0.0031	0.0001	0.0000, 0.0002	0.0011	0.0005, 0.0017
Distilled grape marc	Intercept	0.3538	0.3435, 0.3644	0.0201	0.0185, 0.0217	0.3677	0.337, 0.3994
	time	0.0003	−0.0003, 0.0009	0.0000	−0.0001, 0.0001	0.002	0.0011, 0.003
Coffee grounds	Intercept	0.3431	0.3191, 0.3671	0.0267	0.0237, 0.0297	0.4553	0.4097, 0.5007
	time	0.0036	0.0022, 0.0049	0.0003	0.0002, 0.0004	0.0002	−0.0009, 0.0013
Scotch broom	Intercept	0.4431	0.277, 0.6107	0.0322	0.0156, 0.0484	0.4352	0.3713, 0.4983
	time	0.0065	0.0011, 0.0065	0.0003	0.0000, 0.0006	0.0008	−0.0001, 0.0016
Silver wattle	Intercept	0.2781	0.2345, 0.3193	0.0243	0.0156, 0.0332	0.5304	0.5113, 0.5497
	time	0.0105	0.0087, 0.0125	0.0006	0.0003, 0.0009	0.0003	−0.0003, 0.0008
Seaweeds	Intercept	0.3223	0.2800, 0.3675	0.0217	0.0155, 0.0282	0.3865	0.3549, 0.4183
	time	0.0183	0.0141, 0.0221	0.0018	0.0012, 0.0024	0.0056	0.004, 0.0072

Vermicomposting also led to an increase in functional dissimilarity over time, with turnover rates that were characteristic of each substrate (Fig 2b). Thus, vermireactors fed with silver wattle and seaweed showed steeper slopes than other vermireactors, which implies 3–9 times faster rates of temporal turnover of functional dissimilarity (Fig 2b, S4 Fig, Table 2, S1 Table). The baseline functional dissimilarity modeled by the intercept differed between vermicomposting substrates, being 1.6 and 1.3 times higher in vermireactors fed with Albariño grape marc and scotch broom, respectively, compared to other substrates (Fig 2b, Table 2, S1 Table). Temporal turnover for functional composition was statistically significant for all slopes except for the vermireactor fed with distilled grape marc (Table 2, S1 Table). When analysing all substrates together the overall temporal turnover rate decreased to values similar to those of vermireactors fed with Albariño grape marc, coffee grounds and scotch broom (time estimate = 0.0003, 95% CI = 0.0002, 0.0004), with a baseline dissimilarity value of 0.0404 (95% CI = 0.0127, 0.0677).

Vermicomposting led to increasing functional redundancy of bacterial communities, although differed across vermireactors (Fig 2c, S5 Fig). The vermireactor fed with seaweed had the steepest slope, resulting in a 3 and 13 times higher temporal change compared to vermireactors fed with the three grape marcs and the other three substrates, respectively (Fig 2c, Table 2, S1 Table). Moreover, the vermireactor fed with Albariño grape marc showed the highest baseline level of functional redundancy, which was 1.3 to 1.7 times higher than those of the other vermireactors. All slopes except the slopes of vermireactors fed with coffee grounds, scotch broom, and silver wattle, were statistically significant (Table 2, S1 Table). When analysing all substrates together the overall temporal turnover rate decreased to values similar to those of vermireactors fed with Albariño and distilled grape marc (time estimate = 0.00241, 95% CI = 0.0019, 0.0029), with a baseline dissimilarity value of 0.306 (95% CI = 0.285, 0.327).

## Discussion

Our analyses of 16S rRNA data in seven vermireactors revealed that vermicomposting leads to significant and related changes in both taxonomic and functional bacterial communities. This is evident even without comparing our data against control samples without earthworms, as showed in taxonomic and functional PCoAs (Fig 1 and S1 and S2 Figs). One limitation of our study is that, because only 16S rRNA amplicon data were collected, microbial functions could only be predicted from taxonomic composition rather than estimated directly. However, previous studies have shown that PICRUSt2 functional predictions strongly correlate with shotgun metagenomic functional profiles (mean correlation > 0.8 across diverse sample types [23]), supporting its use as a reliable tool for functional inference. A potential limitation of this study is the absence of a control treatment without earthworms. Consequently, our findings reflect the combined, synergistic activity of earthworms and microorganisms, which defines vermicomposting in the strictest sense. While this approach captures the full dynamics of the system and prevents us from isolating the independent effects of the earthworms, the inclusion of diverse earthworm densities and contrasting substrates ensures the robustness and broad generalizability of our findings across different vermicomposting substrata. Similar results, i.e., functional changes mirrored by taxonomic changes, have been reported in other microbial communities including reactors of wastewater treatment plants [33], soils [34–36], acid mine drainages [37], ocean waters [38,39] and different vermicomposting systems [3,9–14,40]. However, none of those studies specifically examined turnover rates in taxonomic and functional composition, which is the main focus of the present work. During the final stages of vermicomposting, we did not observe a clear separation of samples based on their function, as was observed for their taxonomic composition. This indicates an increasing amount of functional redundancy over time in the vermicomposting process, as occurred in most vermireactors. On the other hand, unsegregated patterns in the PCoA plots over the whole vermicomposting process are indicative of lesser functional redundancy, as occurred with the silver wattle vermireactor. In the case of the vermireactor fed with coffee grounds, the samples were slightly more segregated, resulting in increased functional redundancy over time.

Our data showed that the turnover rate differed greatly between taxonomic and functional composition with taxonomic turnover being 12 times higher overall. This, again, highlights the significant functional redundancy observed in these vermicomposting systems. Our study found that vermireactors fed with seaweed and silver wattle had the highest rates of turnover for both taxonomic and functional composition. The two differed not only in their processing times (28 and 56 days, respectively), but also in their overall earthworm population density (850 and 640 earthworms m^-2^, respectively). Since GAPs are density-dependent, as previously shown with coarser microbiological analysis [8], we would expect the vermireactors with the highest earthworm densities to have the highest rates of both taxonomic and functional composition turnover (spent coffee grounds, silver wattle, and distilled grape marc) or the shortest processing times (seaweed, distilled grape marc, spent coffee grounds and silver wattle). Accordingly, those vermireactors showed the highest taxonomic and functional turnover rates, except the vermireactors fed with distilled grape marc and coffee grounds; this could be due to the low alpha-diversity variation observed in those vermireactors [11].

Our research shows that vermicomposting generates significant changes over time in the taxonomic and functional composition of bacterial communities. Initially, non-linear temporal trends arise due to the abrupt microbial transition from raw substrates to the first stages of earthworm activity (i.e., GAPs). However, once CAPs were implemented, a temporal linear trend dominates both taxonomic and functional composition turnovers. The variance explained by PERMANOVA and PCoA axes was notably higher than reported in the literature for soil bacteria succession over time [7,41–43], the phyllosphere [44–46], or the microbiome during animal development [47–50]. This, again, highlights the dramatic changes vermicomposting exerts on taxonomic and functional composition.

In this study we did not delve into a detailed analysis of what genes or metabolic pathways were enriched during vermicomposting. We showed that vermicomposts have increased gene contents in pathways related with either plant growth (biosynthesis of amino acids, plant hormone synthesis or nitrogen metabolism) or environmental functions (furfural and bisphenol degradation and antibiotic synthesis) [3,9–14]. Functional redundancy is positively correlated with ecosystem stability and resiliency [51], and microbial diversity is a good predictor of ecosystem functioning [52]. Vermicomposts studied here (see references above) have not only higher functional gene content but also higher microbial diversities than those of raw substrates. Therefore, extending the vermicomposting process should result in a more stable and resilient product.

## Conclusions

While previous research has established vermicomposting as a viable method for transforming waste into organic amendments, the temporal dynamics and density dependence of taxonomic and functional bacterial shifts have remained unclear. Our results reveal significantly higher turnover rates for both taxonomic and functional composition (0.006 and 0.0004 Sørensen units per day, respectively) than reported for soil bacteria (0.000027 Sørensen units per day for taxonomic composition [7]). The higher taxonomic relative to functional turnover suggests substantial functional redundancy during microbial succession, a pattern consistent across all substrates and reactors. Although functional redundancy increased over time, the distinct functionalities retained within different bacterial communities indicate that their application as organic amendments should be carefully monitored. Additionally, minor differences in turnover rates may reflect variation in earthworm densities across substrates. Future studies that include a broader range of vermicomposting substrates, such as animal manures or sewage sludge, would help determine whether these turnover patterns are a general feature of vermicomposting. Because this study lacked a non-earthworm control, the observed accelerated turnover rates cannot be attributed solely to earthworm activity, but rather to the combined effects of microorganisms and earthworms. Future research should address this limitation by incorporating non-worm controls to isolate the specific contribution of earthworms and by examining the effects of varying earthworm densities. Finally, given the established links between microbial diversity and ecosystem functioning, as well as between functional redundancy and ecosystem stability and resilience, our findings suggest that prolonging the vermicomposting process may yield a more stable and resilient end product.

## Supporting information

S1 Fig PCoA ordination plots of taxonomic composition of bacterial communities during vermicomposting of white (Albariño), red (Mencía) and distilled grape marc, spent coffee grounds, scotch broom and silver wattle. (PDF)

S2 Fig PCoA ordination plots of functional composition of bacterial communities during vermicomposting of white (Albariño), red (Mencía) and distilled grape marc, spent coffee grounds, scotch broom and silver wattle. (PDF)

S3 Fig Patterns of temporal changes in taxonomic composition of bacterial communities during vermicomposting of white (Albariño), red (Mencía) and distilled grape marc, spent coffee grounds, scotch broom, silver wattle and seaweed. Lines show the mixed-model fit. Note that each vermicomposting reactor has different measures of time. The intercepts represent the baseline community dissimilarity when temporal distance equals zero, and the slopes represent the overall rates of community turnover.
(PDF)

S4 Fig Patterns of temporal changes in functional composition of bacterial communities during vermicomposting of white (Albariño), red (Mencía) and distilled grape marc, spent coffee grounds, scotch broom, silver wattle and seaweed. Lines show the mixed-model fit. Note that each vermicomposting reactor has different measures of time. The intercepts represent the baseline community dissimilarity when temporal distance equals zero, and the slopes represent the overall rates of community turnover.
(PDF)

S5 Fig Patterns of temporal changes in functional redundancy of bacterial communities during vermicomposting of white (Albariño), red (Mencía) and distilled grape marc, spent coffee grounds, scotch broom, silver wattle and seaweed. Lines show the mixed-model fit. Note that each vermicomposting reactor has different measures of time. The intercepts represent the baseline functional redundancy when time equals zero, and the slopes represent the overall rates of functional redundancy turnover.
(PDF)

S1 Table Conditional and marginal R^2^ coefficient for mixed models showed in Table 2. Marginal R^2^ represents the variance explained by the fixed effects. Conditional R^2^ represents the variance explained by the entire model, including both fixed and random effects.
(XLSX)
