# Supplementary material for: Faster turnover of taxonomic over functional bacterial composition during vermicomposting indicates increasing functional redundancy
Source: PLoS One. 2026 Jul 22;21(7):e0354276. doi: 10.1371/journal.pone.0354276 (PMC13390840; doi:10.1371/journal.pone.0354276)
Supplement: S1 Fig — (PDF) [file pone.0354276.s001.pdf]

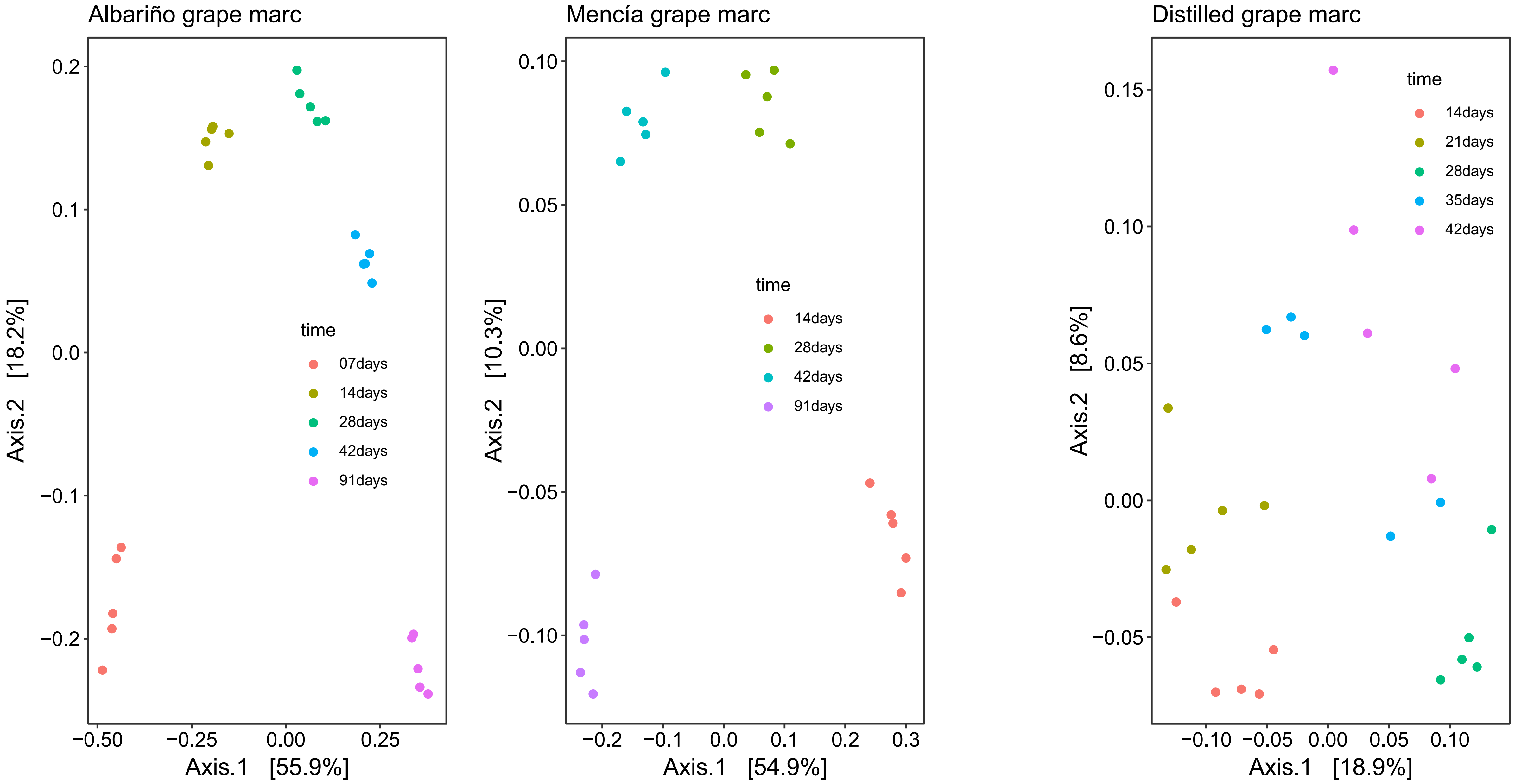

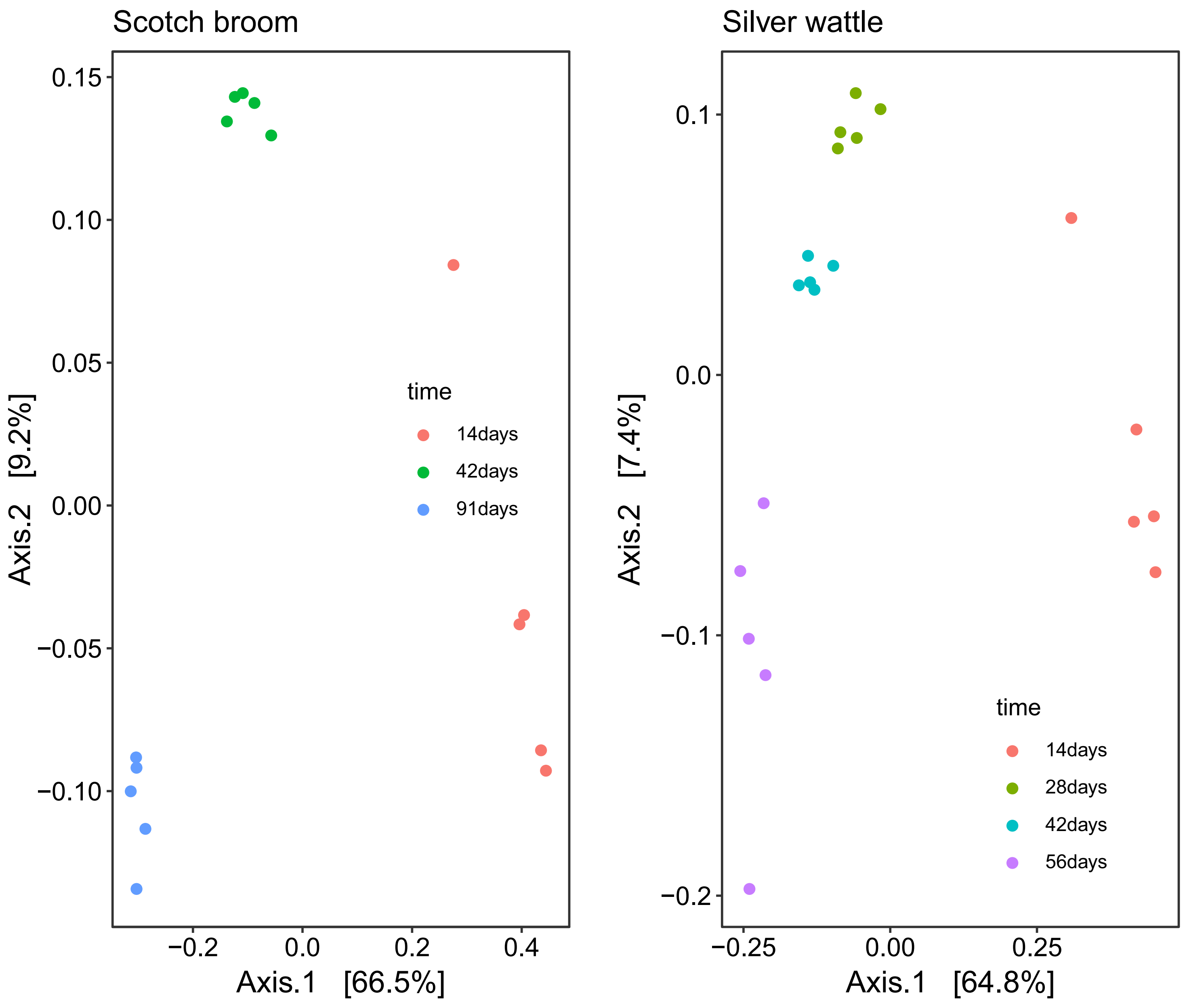

Supplementary Figure 1. PCoA ordination plots of taxonomic composition of bacterial communities during vermicomposting of white (Albariño), red (Mencía) and distilled grape marc, spent coffee grounds, scotch broom and silver wattle
