# Supplementary figures and images for: Faster turnover of taxonomic over functional bacterial composition during vermicomposting indicates increasing functional redundancy
Source: PLoS One. 2026 Jul 22;21(7):e0354276. doi: 10.1371/journal.pone.0354276 (PMC13390840; doi:10.1371/journal.pone.0354276)

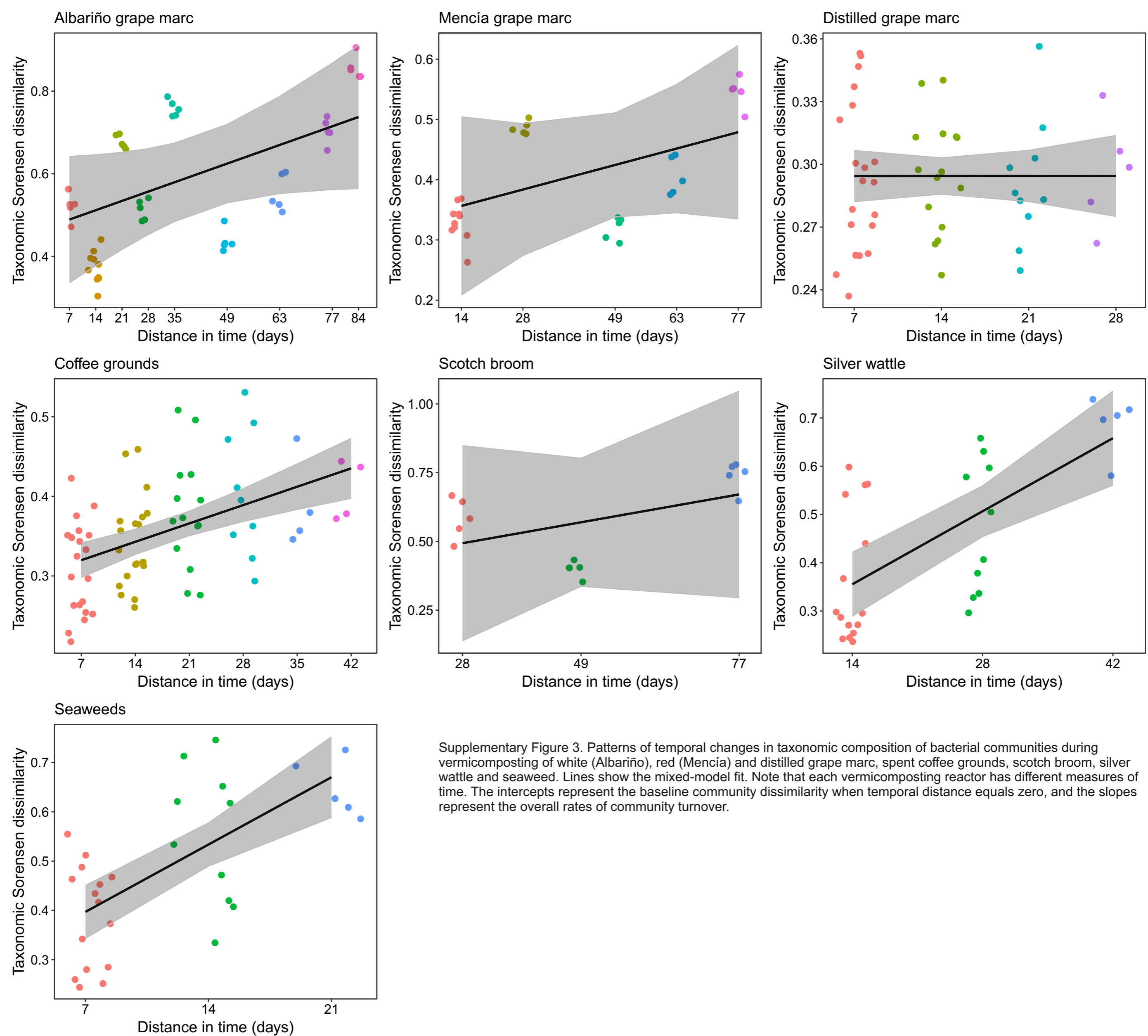

Supplement: S3 Fig — Lines show the mixed-model fit. Note that each vermicomposting reactor has different measures of time. The intercepts represent the baseline community dissimilarity when temporal distance equals zero, and the slopes represent the overall rates of community turnover. (PDF) [file pone.0354276.s003.pdf]

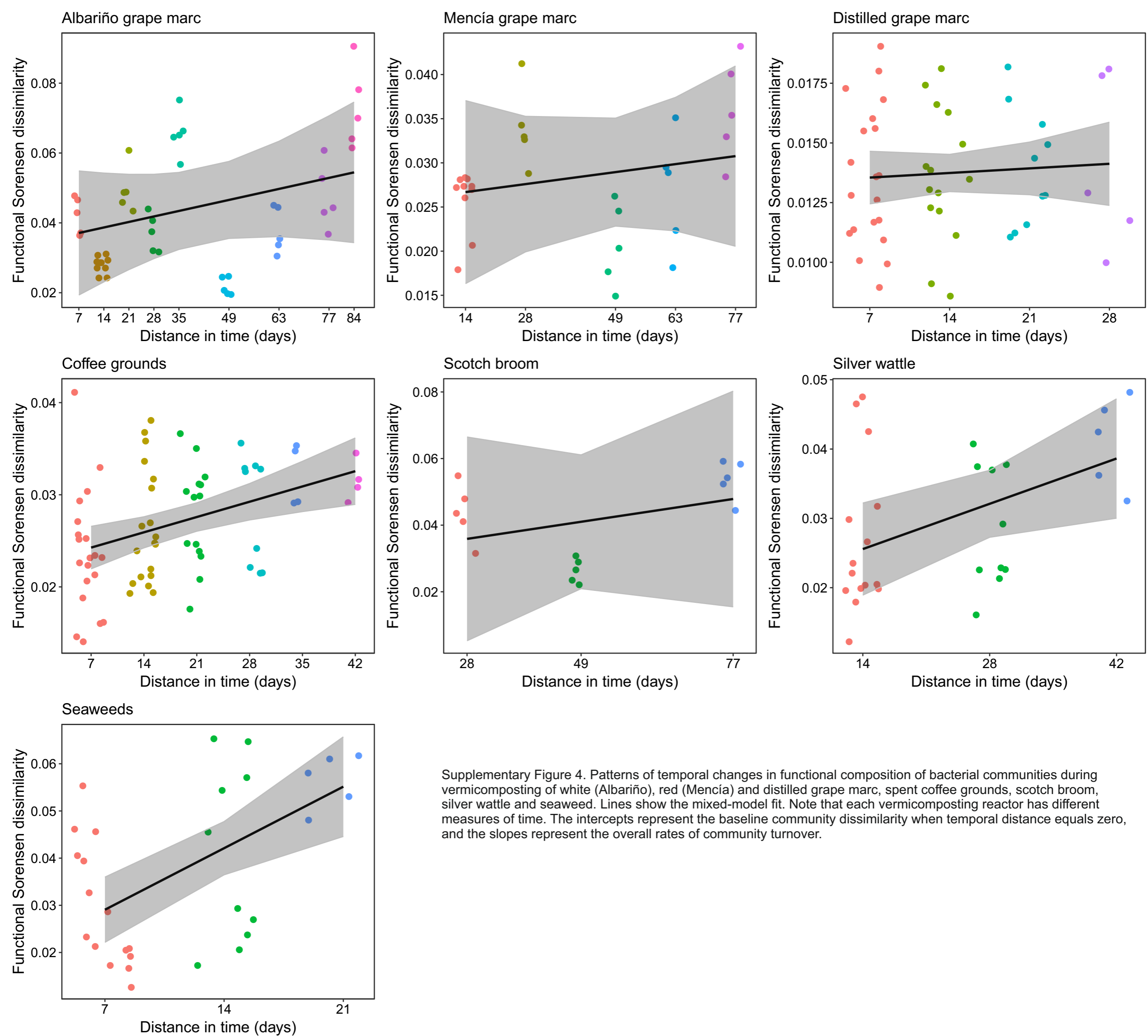

Supplement: S4 Fig — Lines show the mixed-model fit. Note that each vermicomposting reactor has different measures of time. The intercepts represent the baseline community dissimilarity when temporal distance equals zero, and the slopes represent the overall rates of community turnover. (PDF) [file pone.0354276.s004.pdf]

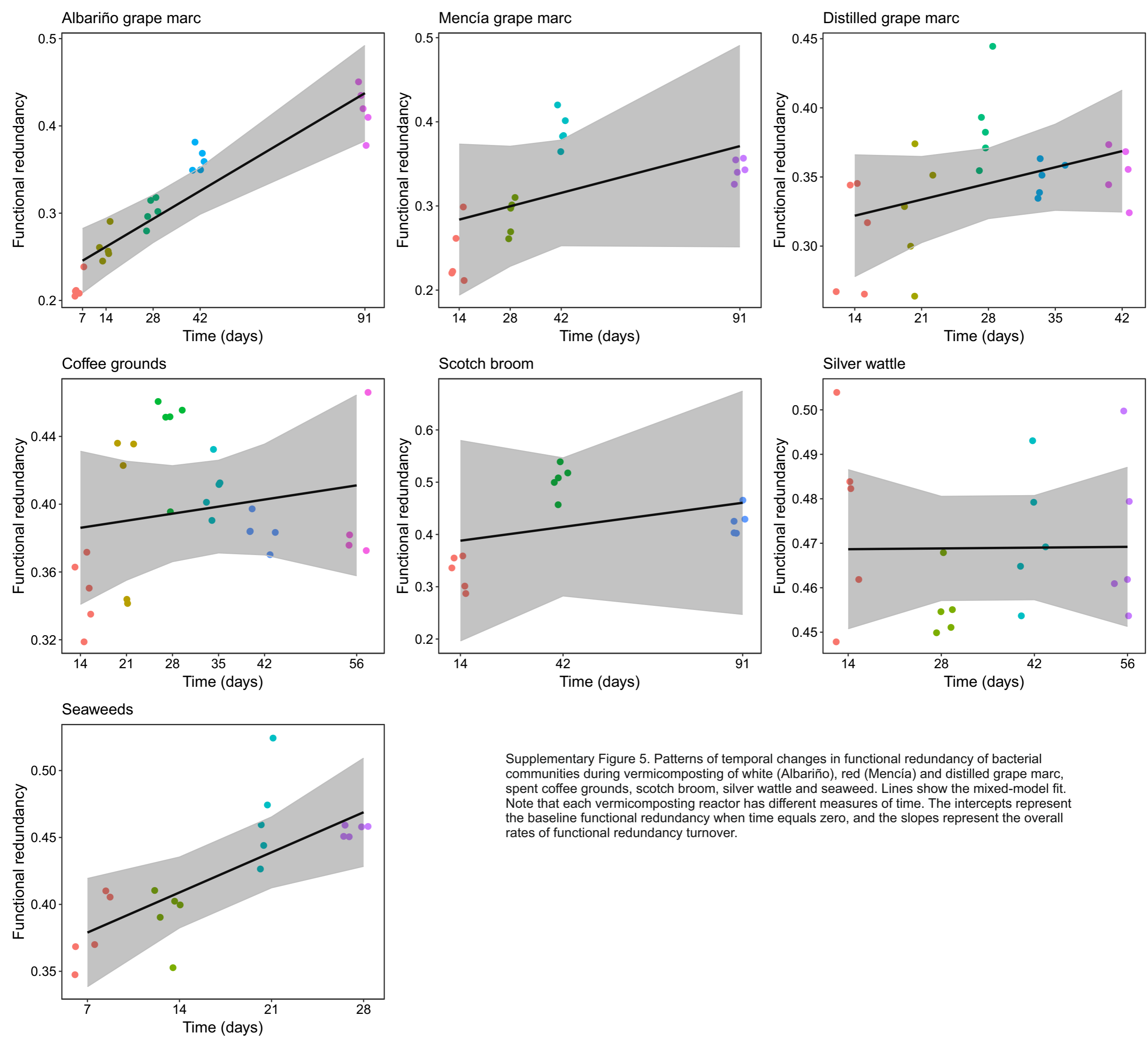

Supplement: S5 Fig — Lines show the mixed-model fit. Note that each vermicomposting reactor has different measures of time. The intercepts represent the baseline functional redundancy when time equals zero, and the slopes represent the overall rates of functional redundancy turnover. (PDF) [file pone.0354276.s005.pdf]
